# Medida do PETCO2 no Limiar Anaeróbico: Melhor Marcador Prognóstico em Pacientes com Ressincronizador?

**DOI:** 10.36660/abc.20220477

**Published:** 2022-08-25

**Authors:** Anderson Donelli da Silveira, Maurício Pimentel

**Affiliations:** 1 Hospital de Clínicas de Porto Alegre Porto Alegre RS Brasil Hospital de Clínicas de Porto Alegre, Porto Alegre, RS - Brasil

**Keywords:** Ergoespirometria/métodos, Volume Sistólico, Terapia de Ressincronização Cardíaca/métodos, Insuficiência Cardíaca, Débito Cardíaco Elevado, Prognóstico

O teste cardiopulmonar de exercício (TCPE) é uma ferramenta consolidada na avaliação funcional e prognóstica do paciente com insuficiência cardíaca^1^ com fração de ejeção reduzida (ICFER), tratando-se de uma pedra angular na avaliação para indicação de terapias avançadas na ICFER.^1 , 2^ A terapia de ressincronização cardíaca (TRC), além de redução da mortalidade, pode melhorar a aptidão cardiorrespiratória, levando a um aumento do consumo de oxigênio de pico (VO_2_ pico) e uma redução da inclinação da relação do equivalente respiratório de CO2 (VE/VCO2 slope).^3 - 5^ Nos últimos anos, com a evolução dos tratamentos, a mortalidade geral e o risco de morte súbita dos pacientes com ICFER vem sendo reduzidas.^6 , 7^ Neste contexto, torna-se importante revisar o prognóstico e os valores associados a um maior risco dentre as variáveis do TCPE nos pacientes submetidos à TRC.

A medida da pressão de dióxido de carbono no final de expiração (P_ET_CO_2_) durante o TCPE, tanto em repouso,^8^ quanto no primeiro limiar ventilatório ou limiar anaeróbio (P_ET_CO_2LA_)^9^ tem valor prognóstico já consolidado na insuficiência cardíaca.^8 , 9^ O aumento da ventilação de espaço morto, causada pelo prejuízo da relação ventilação/perfusão (V/Q) como, por exemplo, nos pacientes com disfunção do ventrículo esquerdo, leva à uma redução do CO2 alveolar e consequentemente do P_ET_CO_2_. É esperado que haja um aumento na sua medida até o limiar anaeróbico e este se correlaciona a um aumento do débito cardíaco.

Neste número dos Arquivos Brasileiros de Cardiologia, Reis et al.,^10^ apresentam interessante análise do papel prognósticos do TCPE em uma coorte de 450 pacientes, sendo 114 submetidos à TRC.^10^ Os pacientes foram seguidos por 2 anos e o desfecho avaliado foi mortalidade cardiovascular e necessidade de transplante urgente. Os estudos clássicos de avaliação para transplante cardíaco^9 , 11^ envolvendo o TCPE não contemplam pacientes com TRC, o que torna importante o questionamento sobre como seria o comportamento das variáveis prognósticas nesse contexto.^9 , 11^ O conhecimento desse cenário é escasso, e algumas evidências sugerem um papel não tão importante do VO_2_ pico nesses pacientes na seleção para transplante cardíaco.^12^

No estudo de Reis et al.,^10^ o VO_2_ pico, o VE/VCO2 slope, o P_ET_CO_2_ e o P_ET_CO_2LA_ apresentaram capacidade de predizer desfechos nos pacientes com IC e TRC, nas análises uni e multivariável.^10^ Todavia, na análise da curva ROC, o P_ET_CO_2LA_ aparentemente apresentou acurácia superior para predição de eventos. De maneira interessante, o ponto de corte ótimo foi de 33 mmHg, mais baixo do que os 36 mmHg de estudos prévios que avaliaram essa variável.^8 , 13^ Já os pontos de corte do VO_2_ pico (12 ml/kg/min) e do VE/VCO2 slope (35), foram semelhantes a valores descritos previamente na literatura^16^, não havendo diferença nos pacientes com e sem TRC.^14^ É importante ressaltar que, nesse estudo observacional, não houve diferença na incidência de eventos cardiovasculares maiores entre os pacientes com e sem TRC. Outro achado importante foi o excelente valor preditivo negativo (VPN) de medidas de PETCO2, VO2pico e VE/VCO2 slope nos seus pontos de corte, mais evidente ainda nos pacientes do grupo TRC.

Uma possível limitação do estudo, já mencionada pelos autores, é a presença de testes submáximos em uma quantidade razoável de pacientes, o que reduz o poder discriminatório do VO_2_ pico para a predição de eventos. Entretanto, as evidências fornecidas, além de consolidar o papel prognóstico do TCPE nesses pacientes, chamam atenção para a importância da medida do P_ET_CO_2LA_ rotineiramente nesses casos. O uso dessa variável apresenta excelente poder prognóstico e pode adicionar informações às medidas tradicionais do TCPE. A sua medida apresenta excelente relação com o aumento do débito cardíaco ao esforço, e uma resposta alterada (ausência de aumento até o LA) caracteriza maior prejuízo no aumento do débito no exercício.

Estudos com maior tamanho amostral, preferencialmente multicêntricos, avaliando o poder prognóstico do P_ET_CO_2LA_ nos pacientes com IC e comparando-o às outras medidas prognósticas são bem-vindos. A replicação de resultados em populações distintas fortalece a evidência encontrada e amplia a validade externa dos achados encontrados.
