# Supplementary material for: Medida do PETCO2 no Limiar Anaeróbico: Melhor Marcador Prognóstico em Pacientes com Ressincronizador?
Source: Arq Bras Cardiol. 2022 Aug 25;119(3):424–5. [Article in Portuguese] doi: 10.36660/abc.20220477 (PMC9438545; doi:10.36660/abc.20220477)
Supplement: Translation

# Translation: Measurement of PETCO2 at Anaerobic Threshold: A Best Prognostic Marker in Patients with Cardiac Resynchronization Therapy?

**Authors:** Anderson Donelli da Silveira, Maurício Pimentel

**Affiliations:** 1 Brazil Hospital de Clínicas de Porto Alegre, Porto Alegre, RS - Brazil

**Keywords:** Ergoespyrometry/methods, Stroke Volume, Cardiac Resynchronization Therapy/methods, Heart Failure, Cardiac Output, High, Prognosis

The cardiopulmonary exercise test (CPET) is a consolidated tool in the functional and prognostic assessment of patients with heart failure^1^ with reduced ejection fraction (HFrRF), being a cornerstone in the evaluation for the indication of advanced therapies in HFrEF.^1 , 2^ Cardiac resynchronization therapy (CRT), in addition to reducing mortality, can improve cardiorespiratory fitness, leading to an increase in peak oxygen consumption (VO_2_peak) and a reduction in the slope of the respiratory equivalent CO2 ratio (VE /VCO2 slope).^3 - 5^ In recent years, with the evolution of treatments, general mortality and the risk of the sudden death of patients with HFpEF have been reduced.^6 , 7^ In this context, reviewing the prognosis and the values associated with a higher risk among the CPET variables in patients undergoing CRT becomes important.

The measurement of end-tidal carbon dioxide pressure (PETCO2) during CPET, both at rest,^8^ and at the first ventilatory threshold or anaerobic threshold (P_ET_CO2_LA_),^9^ has a well-established prognostic value in heart failure.^8 , 9^ The increase of dead space ventilation, caused by the impairment of the ventilation/perfusion (V/Q) ratio, for example, in patients with left ventricular dysfunction, leads to a reduction in alveolar CO2 and, consequently, in P_ET_CO_2_. It is expected that there will be an increase in its measurement up to the anaerobic threshold, which is correlated with an increase in cardiac output.

In this issue of *Arquivos Brasileiros de Cardiologia* , Reis et al.^10^ present an interesting analysis of the prognostic role of CPET in a cohort of 450 patients, 114 of whom underwent CRT.^10^ The patients were followed up for 2 years, and the evaluated outcome was cardiovascular mortality and the need for urgent transplantation. The classic evaluation studies for heart transplantation^9 , 11^ involving CPET do not include patients with CRT, which makes it important to question how the behavior of prognostic variables would be in this context.^9 , 11^ Knowledge of this scenario is scarce, and some evidence suggests a less important role of peak VO_2_in these patients in the selection for heart transplantation.^12^

In the study by Reis et al.,^10^ VO_2_peak, VE/VCO2 slope,_PET_CO2 and P_ET_CO2_LA_were able to predict outcomes in patients with HF and CRT in uni- and multivariable analyses.^10^ However, in the ROC curve analysis, P_ET_CO2_LA_apparently showed superior accuracy for predicting events. Interestingly, the optimal cut-off point was 33 mmHg, lower than the 36 mmHg in previous studies that evaluated this variable.^8 , 13^ On the other hand, the cut-off points for peak VO_2_(12 ml/kg/min) and VE/VCO2 slope (35) were similar to values previously described in the literature16, with no difference in patients with and without CRT.^14^ It is important to emphasize that, in this observational study, there was no difference in the incidence of major cardiovascular events between patients with and without CRT.

A possible limitation of the study, already mentioned by the authors, is the presence of submaximal tests in a reasonable number of patients, which reduces the discriminatory power of peak VO^2^ for predicting events. However, the evidence provided, in addition to consolidating the prognostic role of CPET in these patients, calls attention to the importance of routinely measuring P_ET_CO2_LA_in these cases. This variable has excellent prognostic power and can add information to traditional CPET measurements. Its measurement has an excellent relationship with the increase in cardiac output on exertion, and an altered response (absence of increase up to the AT) characterizes a greater loss in the increase in output during exercise.

Studies with larger sample sizes, preferably multicenter, evaluating the prognostic power of P_ET_CO2_LA_in patients with HF and comparing it to other prognostic measures are welcome. The replication of results in different populations strengthens the evidence found and expands the external validity of the findings.
